# Reduction in Door-to-Needle Time after Transfer of Thrombolysis Site from CCU to Emergency Department

**DOI:** 10.1155/2013/208271

**Published:** 2013-09-24

**Authors:** Osama Mohammed, Firjith C. Paramba, Naushad V. Aboobaker, Riyadh A. Mohammed, Nishan K. Purayil, Haitham M. Jassim, Mohammad K. Shariff, Saud M. Aslam, Farook F. Muhsen, Khalid H. Al Noor, Hani H. Al Kilani

**Affiliations:** Department of Emergency Medicine, Al Khor Hospital, Hamad Medical Corporation, P.O. Box 21086, Doha, Qatar

## Abstract

*Objective*. Early restoration of coronary perfusion by thrombolysis or percutaneous coronary intervention is the main modality of treatment to salvage the ischemic myocardium. The earlier the procedure is completed, the greater the benefit is in saving myocardium and restoring its functions. The aim of the study is to compare the door-to-needle time (DNT) in acute ST elevation myocardial infarction (STEMI) in the period prior to December 2008 when the site of thrombolysis was in coronary care unit (CCU) and the period after that when the site was shifted to emergency department (ED). *Methods*. A retrospective, descriptive study was conducted at Al Khor Hospital, Qatar, in patients with acute STEMI who underwent thrombolysis at CCU and ED from April 2005 until December 2011, to compare the DNT, duration of hospitalization, and mortality. *Results*. A total of 211 patients with acute STEMI were eligible for thrombolysis; 58 patients were thrombolysed in the CCU and 153 in ED. The median DNT was reduced from 33.5 minutes in the CCU to 17 minutes in the ED representing a reduction of more than 50% with a *P* value of < 0.0001. *Conclusion*. The transfer of the thrombolysis site from CCU to the ED was associated with a dramatic and significant reduction in median door-to-needle time by more than half.

## 1. Introduction

 Acute reperfusion therapy performed either with thrombolytic therapy or percutaneous coronary intervention (PCI) is the mainstay of treatment for patients with acute ST-segment elevation myocardial infarction (STEMI). The benefit of the perfusion is restoring coronary flow which is time-dependent; the earlier the reperfusion is established, the greater the benefit is in saving the myocardium [1–3]. Randomized clinical trials have shown that early reperfusion therapy reduces the overall 30-day mortality by 17–25%, with increasing benefit as the time from onset of pain to the initiation of thrombolytic therapy is reduced [4–6]. Since symptoms-to-door time (SDT) is beyond the control of the medical team in the hospital, the focus is stressed on decreasing the time from the first medical contact to reperfusion therapy in acute myocardial infarction. Hence, the importance of door-to-needle time (DNT) for thrombolytic therapy and door-to-balloon time for PCI has emerged. These interventions occur within the hospital and can be controlled with proper training of the medical and nursing staff and by applying international practice guidelines. DNT is the time taken from patient's arrival to a medical facility to the time when thrombolytic therapy is administered. As a result of the importance of the timing of the thrombolysis, DNT time has emerged as an important hospital performance measure for the quality of care of patients with STEMI in the United States and Europe [6–8]. 

 The American College of Cardiology/the American Heart Association (ACC/AHA) and the European Society of Cardiology (ESC) guidelines for STEMI recommend that the DNT for thrombolysis should be within 30 minutes of first medical system contact [6, 7]. Hospitals fail to achieve this goal because of the fact that thrombolytic therapy is often not initiated in the ED [9–13]. In some hospitals therapy may be initiated in the ED, but only a minority of patients are thrombolysed within this time frame because of varying ED protocols [4, 5, 14, 15]. Hence we decided to conduct a study comparing the DNT of thrombolysis in CCU versus ED in patients presenting with acute STEMI.

## 2. Patients and Methods

**Figure 1:**
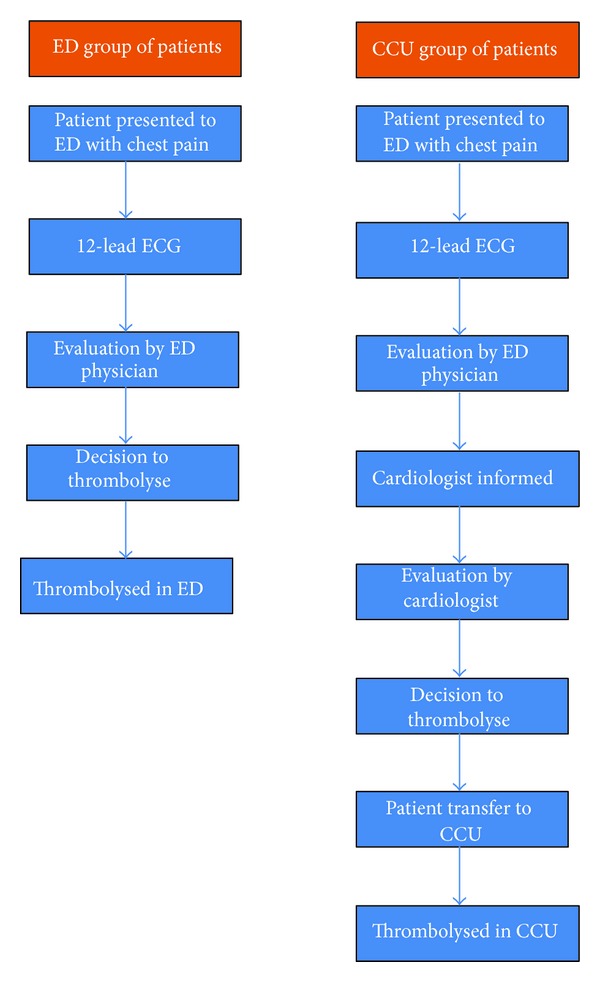
Flow chart showing the process of thrombolysis in ED and CCU group.

This study was carried out at the ED, Al-Khor Hospital, Hamad Medical Corporation, Qatar. Prior to December 2008, patients presenting with acute STEMI to ED were thrombolysed in the coronary care unit (CCU) after evaluated by the cardiologist. A well-trained team comprising of ED physicians and nurses was formed in the year January 2009 for thrombolysing acute STEMI patients in ED. Patients who were diagnosed of having acute STEMI were evaluated by ED physician and subsequently thrombolysed in the ED (Figure 1). Door-to-needle time for thrombolysis in this group were compared with those of patients who were thrombolysed in CCU prior to 2009. A retrospective data collection was made from the medical records and computerized departmental data base. This included demographic features, comorbid conditions, time of onset of symptoms, time of arrival to hospital, indication for thrombolysis, door-to-needle time, symptom-to-door time, and course in the hospital which were all noted. Patients with delayed presentation and incomplete medical records and who presented with an initial nondiagnostic ECG were excluded. The study was approved by the Ethics Committee of the medical research department (approval number #10170/10).

## 3. Statistical Analysis

Categorical and continuous values are expressed as frequency, percentage, mean ± SD, median, and range. Descriptive statistics were used to summarise all demographic and other clinical characteristics of the patients. Quantitative variables means for the two thrombolysis sites (independent groups) were analyzed using the unpaired *t*-test. For nonnormal data (skewed), the corresponding nonparametric Mann-Whitney *U* test was applied to assess significant difference in DNT between the two thrombolysis sites (CCU and ED). Associations between two or more qualitative or categorical variables were assessed using Chi-square tests. Pictorial representations of the key results were made using appropriate statistical graphs, including box plot and bar diagrams. A two-sided *P*  value < 0.05 was considered as statistically significant. All statistical analyses were conducted using IBM SPSS statistical package V19 (SPSS Inc., Chicago, IL). 

## 4. Results

**Figure 2:**
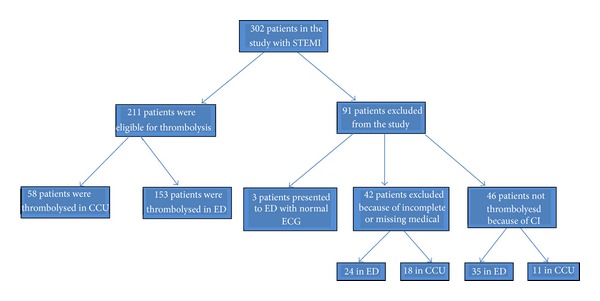
Flow chart of patient allocation.

A total of 302 acute STEMI patients were included in the analysis out of which 211 patients with acute STEMI were eligible for thrombolysis: 153 in the ED and 58 in the CCU. Ninety-one patients were excluded from the study. Details are shown in Figure 2. The base line characteristics and site of infarctions of both the groups are shown in Table 1. There were no significant differences between the two groups. The majority of the patients were men, and most commonly comorbid conditions were hypertension, diabetes, and dyslipidemia.

**Figure 3:**
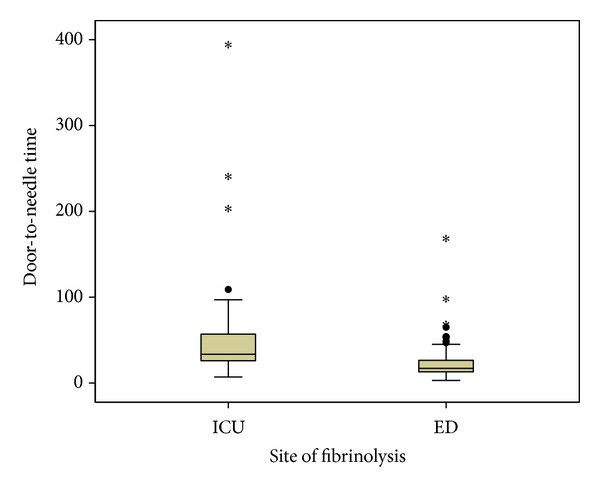
Box plot graph comparing DNT in both the ED and the CCU patient groups. CCU: coronary care unit and ED: emergency department.

There was a significant reduction in the DNT of patients thrombolysed in ED. The mean DNT was 17 minutes in the ED group compared to 33.5 minutes in the CCU group, representing more than 50% reduction in DNT with a *P*  value  of < 0.0001 (Table 2, Figure 3). The SDT was 120 minutes in both the groups. Mean duration of hospitalization (days) did not differ significantly between the CCU and ED groups (5.3 ± 1.8 and 5.7 ± 2.1; *P*  value = 0.155). One patient from the CCU group and 2 from the ED group died during treatment. Two patients from the ED group were thrombolysed according to the ED STEMI protocol, but in both patients, cardiac enzymes remained normal and there was no regional wall abnormality on the Echocardiography. The symptoms were probably due to coronary artery spasm.

## 5. Discussion

Recent advancements in the medical technologies have revolutionized the treatment of acute myocardial infarction. Even though primary PTCA has been the mainstay of treatment in the present era, it is limited to a few tertiary care centers and is not affordable for all the patients especially in the developing world. Hence thrombolysis is still the treatment of choice for reperfusion in most parts of the world [6, 16]. Earlier trials have demonstrated the benefit of giving thrombolysis as early as possible in acute STEMI patients [17–21].

**Figure 4:**
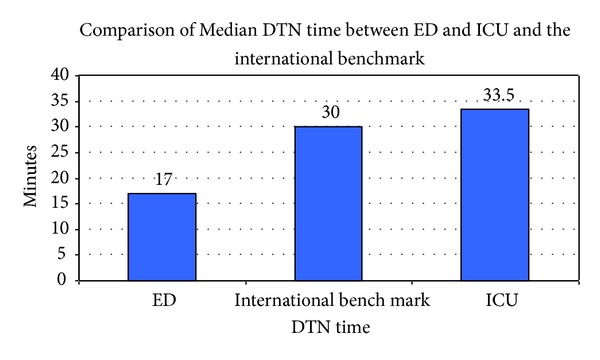


In our study, shifting thrombolysis to ED was associated with a major reduction in the DNT (from 33.5 to 17 minutes), which is well within the international guidelines (Figure 4).

This result was much better than the results reported in previous studies [4–6, 8, 10, 11, 13–15]. Although the DNT reduction was highly significant, there was no significant reduction in hospitalization days or mortality among either of the groups which is similar to what was found in some previous studies [4, 8, 14], while the analysis by the myocardial infarction triage and intervention trial showed a 7-fold reduction of 30 day mortality in patients treated within 70 minutes [22]. Another study showed an independent association between one year mortality and ST-segment elevation in ECG as well as prognostic interaction of time to treatment and ST-segment resolution [23]. 

We have also looked at the safety issues for changing the site of thrombolysis. There was no overall difference in the mortality among both groups. Reducing the DNT has been a challenge for most of the hospitals around the world. Multiple consultations between the primary care physician/ED physician and cardiologist, lack of having trained ED staff, overcrowding of ED, and time taken to transfer the patient to CCU have been given as reasons for delayed DNT. However precious time is saved by having an organised team approach in the ED and accelerating the decision making for thrombolysis.

## 6. Conclusion

 By collaborative and effective team work, thrombolytic therapy can be administered in the emergency department within a short time without compromising the safety of the patient and accuracy of diagnosis. This can be used as a monitoring tool for quality improvement in the ED.

## Figures and Tables

**Table 1:** Demographic and clinical data in patients with acute STEMI.

Variable	CCU group *N* = 58	ED group *N* = 153
Age		
Mean ± SD	46.05 ± 7.47	47.31 ± 7.99
Median (range)	46 (27–73)	47 (25–63)
Gender	Male = 56 (96.55%)	Male = 150 (98.04%)
	Female = 2 (3.45%)	Female = 3 (1.96%)
Hypertension	13 (27%)	31 (28%)
Diabetes	18 (31%)	43 (30%)
High LDL > 3 mmol/L*	25 (46%)	59 (44%)
Smoking	32 (68%)	68 (61%)
Type of MI		
Anterior MI	29 (50%)	66 (43%)
Nonanterior MI	29 (50%)	87 (57%)

*According to the European Society of Cardiology guidelines 2012.

**Table 2:** Door-to-needle time, symptom-to-door time, and duration of hospital stay.

Variable	CCU group	ED group	*P* value
DNT (in minutes)			
Mean ± SD	54.59 ± 64.09	22.20 ± 18.11	<0.0001
Median (range)	33.5 (7–394)	17 (3–168)	
Duration of hospital stay (in days)			
Mean ± SD	5.30 ± 1.84	5.72 ± 2.05	0.155
Median (range)	5 (1–13)	5 (0–16)	
SDT (minutes)			
Mean ± SD	179.52 ± 166.13	149.37 ± 110.67	0.262
Median (range)	120 (20–630)	120 (30–540)	

DNT: Door-to-needle time; SDT: symptom-to-needle time.
